# Cardiac T2* magnetic resonance for prediction of cardiac complications in thalassemia major

**DOI:** 10.1186/1532-429X-11-S1-O2

**Published:** 2009-01-28

**Authors:** Paul Kirk, Michael Roughton, John B Porter, John M Walker, Mark A Tanner, Junaid Patel, Dianne Wu, Jane Taylor, Mark A Westwood, Lisa J Anderson, Dudley J Pennel

**Affiliations:** 1 Royal Brompton Hospital, London, UK; 2 University College Hospital London, London, UK

**Keywords:** Heart Failure, Ferritin, Serum Ferritin, Thalassemia, Thalassaemia Major

## Background

Myocardial siderosis is the main cause of morbidity and mortality in thalassaemia major. In the United Kingdom approximately 50% of patients die before reaching 35 years. The cardiomyopathy is reversible if chelation is commenced early but diagnosis is often delayed due to the late onset of symptoms. T2* CMR can now assess cardiac iron directly and this has profound implications for clinical management of iron overload and the assessment of chelation regimes.

Left ventricular ejection fraction falls with increasing myocardial iron (reduced myocardial T2*; normal value >20 ms), and accordingly iron overloaded patients with symptomatic heart failure have a low T2*. Although data is available on the level of T2* in patients developing heart failure there is no published data on the incidence of heart failure and arrhythmia in patients during follow-up according to baseline myocardial T2*. The aim of this study therefore was to establish the risk of cardiac complications in patients with cardiac siderosis as measured by T2*.

## Methods

A prospective database containing clinical data and T2* values on 652 thalassaemia major patients (1442 scans) was maintained over a 6 year period with 1,285 patient years of prospective follow-up. Of these patients, 319 were male and 333 female with a mean age at time of first scan of 27.1 ± 9.6 years. The mean number of blood units transfused per year per patient was 32.6 ± 11.5.

## Results

At 1 year of follow-up, there were 84 episodes of heart failure and 100 episodes of arrhythmia. There were 4 deaths, with 3 patients dying from sepsis following bone marrow transplant and 1 patient dying following an episode of ventricular tachycardia.

### Heart failure

**Figure 1:**
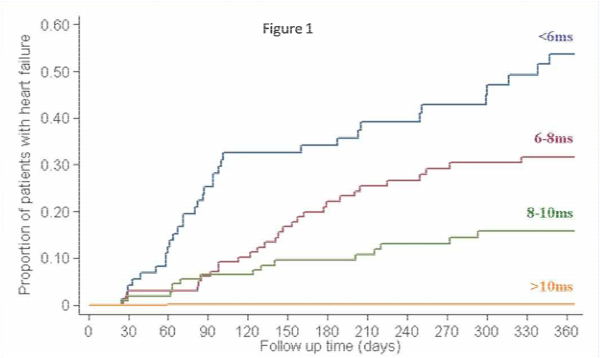
Figure 1

For the 84 heart failure episodes, 64 presented in New York Heart Association (NYHA) class two, 16 were NYHA class three, and 4 were NYHA class four. The mean ejection fraction of these patients was 43.1 ± 7.2%. In these heart failure patients, the preceding cardiac T2* was 6.7 ± 1.8 ms, the liver T2* 3.9 ± 3.7 ms and ferritin 2,713 ± 1,686 μg/L. In comparison with cardiac T2* values >20 ms, there was a significantly increased risk of heart failure associated with cardiac T2* values < 10 ms (Relative Risk 159, P < 0.001) and T2*<6 (RR 268, P < 0.001). Serum ferritin using the conventional threshold was a significant but weaker predictor of heart failure (ferritin >2500 μg/L, RR 0.56, P = 0.021). Liver T2* < 0.96 ms (equivalent to the conventional threshold of >15 mg/g/dw iron) was not a significant predictor of heart failure (liver T2* < 0.96 ms, RR 1.25, P = 0.76). The Kaplan Meier curve of T2* vs heart failure is shown in Figure 1.

### Arrhythmia

**Figure 2:**
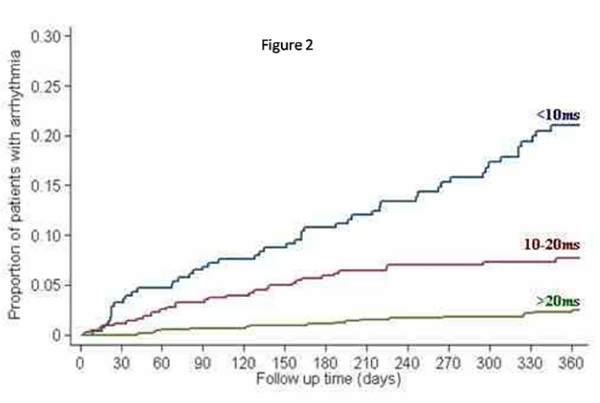
Figure 2

For the 100 episodes of arrhythmia, 79 episodes were atrial fibrillation (AF), 14 episodes were supraventricular tachycardia (SVT), 6 episodes were ventricular tachycardia (VT), and 1 episode was ventricular fibrillation (VF). The mean cardiac T2* was 13.5 ± 9 ms, mean liver T2* 6.0 ± 6.4 ms, mean serum ferritin 2140 ± 1540 μg/L, and the mean ejection fraction was 60.7 ± 9.3%. In comparison with cardiac T2* values >20 ms, there was a significantly increased risk of arrhythmia associated with cardiac T2* values <6 ms (RR 8.65, P < 0.001) and T2*<20 (RR 4.6, P < 0.001). There was no significant predictive value using the conventional thresholds of ferritin (ferritin >2500 μg/L, RR 0.90, P = 0.66) or liver T2* (T2* < 0.96 ms, RR 0.78, P = 0.68). (See Figure 2.)

## Conclusion

These data provide strong evidence that a myocardial T2* <10 ms predicts a high risk of developing heart failure. It is clear that these patients should be aggressively chelated to reduce their high morbidity and mortality from cardiac siderosis.

